# Variability in Physical Inactivity Responses of University Students during COVID-19 Pandemic: A Monitoring of Daily Step Counts Using a Smartphone Application

**DOI:** 10.3390/ijerph19041958

**Published:** 2022-02-10

**Authors:** Shoji Konda, Issei Ogasawara, Kazuki Fujita, Chisa Aoyama, Teruki Yokoyama, Takuya Magome, Chen Yulong, Ken Hashizume, Tomoyuki Matsuo, Ken Nakata

**Affiliations:** 1 Department of Health and Sport Sciences, Graduate School of Medicine, Osaka University, Suita 5650871, Japan; ogasawaraissei@hss.osaka-u.ac.jp (I.O.); fujita@celas.osaka-u.ac.jp (K.F.); aoyama@vision.hss.osaka-u.ac.jp (C.A.); tyokoyam@cardiology.med.osaka-u.ac.jp (T.Y.); t-magome@anat2.med.osaka-u.ac.jp (T.M.); hashizume@skill.hss.osaka-u.ac.jp (K.H.); tmatsuo@skill.hss.osaka-u.ac.jp (T.M.); 2 Department of Sports Medical Biomechanics, Graduate School of Medicine, Osaka University, Suita 5650871, Japan; 3 Center for Education in Liberal Arts and Sciences, Osaka University, Suita 5600043, Japan; chenyulong@celas.osaka-u.ac.jp; 4 Department of Sociology, Otemon Gakuin University, Suita 5678502, Japan

**Keywords:** health promotion, physical activity, academic calendar, online class, clustering analysis, mobile sensing

## Abstract

This study investigated the changes in physical inactivity of university students during the COVID-19 pandemic, with reference to their academic calendar. We used the daily step counts recorded by a smartphone application (iPhone Health App) from April 2020 to January 2021 (287 days) for 603 participants. The data for 287 days were divided into five periods based on their academic calendar. The median value of daily step counts across each period was calculated. A k-means clustering analysis was performed to classify the 603 participants into subgroups to demonstrate the variability in the physical inactivity responses. The median daily step counts, with a 7-day moving average, dramatically decreased from 5000 to 2000 steps/day in early April. It remained at a lower level (less than 2000 steps/day) during the first semester, then increased to more than 5000 steps/day at the start of summer vacation. The clustering analysis demonstrated the variability in physical inactivity responses. The inactive students did not recover daily step counts throughout the year. Consequently, promoting physical activity is recommended for inactive university students over the course of the whole semester.

## 1. Introduction

The coronavirus disease (COVID-19) has severely affected daily life activities around the world. Governments worldwide have enacted restrictive measures to reduce the risk of infection. The restrictions have led to physical inactivity, which increases the risk for non-communicable diseases [1]. Even before the COVID-19 pandemic, physical inactivity had been recognized as a global contributor to the development of chronic non-communicable diseases [2]. The worldwide prevalence of physical inactivity has been reported to be 27.5% across 168 countries between 2002 and 2016 [3]. The COVID-19 pandemic is expected to further accelerate this trend. The influence of COVID-19 on physical activity has been reported and summarized in systematic reviews [1,4]. Accordingly, physical activity has been reported to be reduced across all reviewed populations due to the restrictive measures introduced during COVID-19 [1]. Furthermore, physical activity has been reported to be associated with lower levels of depression and anxiety during COVID-19 [4] and a reduced risk for severe COVID-19 [5,6,7]. Therefore, it is suggested that physical activity should be evaluated and prescribed, if appropriate, during and after the COVID-19 pandemic.

During the COVID-19 pandemic, since university classes moved online, university students lost their opportunities to attend face-to-face classes or extracurricular activities on campus. This trend led to a worldwide decrease in physical activity among university students, as is clearly demonstrated by several surveys [8,9,10,11,12,13]. A study surveyed university students and employees revealed that light physical activity decreased in undergraduate students but not in others (graduate student, faculty members, staff, and administration) [12]. It was speculated that these results can be explained by the fact that before the pandemic, undergraduate students went to campus and walked across campus to attend classes and meet faculty members and administrators at their offices [12]. The reduction in moderate to vigorous physical activity during the pandemic was greater in younger (18–29 years) and older (≥79 years) individuals compared with those who are middle-aged [14]. The physical activity of university students, therefore, was substantially affected by the social restrictions associated with the COVID-19 pandemic, which has been reported to be a cause of increased depression in university students [13].

Daily step counts (sum of step taken per day) have been considered to be an intuitive metric related to health outcomes monitoring with a high degree of temporal resolution [15,16]. The importance of daily step counts has been demonstrated, showing that adults over 40 years old who recorded more than 8000 steps/day showed a lower mortality than those who recorded less than 4000 steps/day [17]. In general, over 10,000 step/day has been recognized as a recommended daily step count [18,19]. The effectiveness of daily step counts recorded through smartphone applications has been recognized [20]. This metric has been used to indicate levels of physical activity during the monitoring of large-scale global trends [21]. The survey with the largest scale of daily step counts used a dataset of 717,527 people across 111 countries, recorded through a smartphone application [21]. Smartphone applications have the advantage of a high penetration rate [22,23], avoiding the need to distribute activity trackers for research. After the COVID-19 pandemic, the sharp decline in daily step counts in each country coincided with the initiation of restrictive measures by their respective governments [24,25,26,27,28,29,30]. The daily step counts gradually recovered during the restrictive measures, after the relaxation of the restrictions, and then increased after their removal of the restrictive measures [24,25,26,29,30]. A survey of daily step counts using a smartphone application can provide real-time information about activity levels on a national or global basis.

Changes in physical activity due to the COVID-19 pandemic have been found to be heterogeneous [31,32,33]. To the best of our knowledge, the heterogeneity of the relationship between decreases in physical activity and the influence of the academic calendar has not yet been made clear. We hypothesized that the physical activity of the university students would be strongly affected by the academic calendar, in addition to government restrictions, because of the shift to online classes. The smartphone-based survey was expected to enable efficient monitoring of the variation in physical activity for university students because most university students already owned smartphones. Therefore, we aimed to investigate the changes in the physical inactivity of university students, occurring in response to the COVID-19 pandemic, and as influenced by the academic calendar.

## 2. Materials and Methods

### 2.1. Data Collection

The study protocol was approved by the observation research ethics review committee of the Osaka University Hospital (code: 19537-2). We followed the ethical recommendations for human research as stipulated by the Declaration of Helsinki. We used a daily step counts dataset recorded from students who took Health and Sport Science classes. An opt-out opportunity was guaranteed for students after the end of the semester. According to our preliminary survey, more than 99% of the university students owned smartphones. The participants were instructed to use their own smartphone devices, and the model of smartphone and version of operating system was not standardized. It was recommended that the students carried their own iPhones with them as much as possible during daily life and exercise (walking and jogging) for monitoring their own physical activity. The participants who used the iPhone and Android devices were recommended to use Apple Health application (Apple Inc., Cupertino, CA, USA) and Google Fit (Google LCC., Mountain View, CA, USA), respectively, for educational purpose. There were 913 valid analyzable records and 25 invalid (unanalyzable broken files) records across participants. Of the 913 valid records, 603 records from the Apple Health application installed on iPhones were used in the study. Google Fit starts to record after installation while Apple Health starts to record automatically. Thus, the Google Fit users could not record their daily step counts before the start of the first semester. Therefore, we adopted only Apple Health data for research purpose. The period to be analyzed was 287 days, from 1 April 2020 to 12 January 2021. If the participant used Apple Health, but it contained a missing day, the data were excluded from data analysis.

Of the 603 records, the number of female and male students was 208 and 395, respectively. The participants included in this dataset were distributed among the 11 undergraduate schools within a national university in Osaka, Japan. For all participants, the class of Health and Sport Science was a required class for graduation taken in the first-year of university. If the class was an elective class, the university student who took this class may have been more active and had greater interest in physical activity than the student who did not take the class, but no such bias may exist in this dataset. Therefore, the participants included in this analyzed dataset are expected to well reflect the variability in physical activity among typical university students.

### 2.2. Data Analysis

The daily step counts were defined as the sum of the steps taken per day. The descriptive statistics were represented by the median, 25% and 75% quantiles for each day across all participants because the daily step count was not normally distributed. The moving averages with 7-day (1 week) windows were used to detect the seasonal changes in daily step counts during the investigation period. Colormaps were used to observe inter-subject variation in the seasonal changes in daily step counts. In these colormaps, rows represent individual data ordered from top to bottom by average daily step counts taken over the entire recording period. The colormaps were then filtered using a two-dimensional moving average, with a kernel (10 individuals × 7 days) to describe seasonal and group trends.

**Figure 1:**
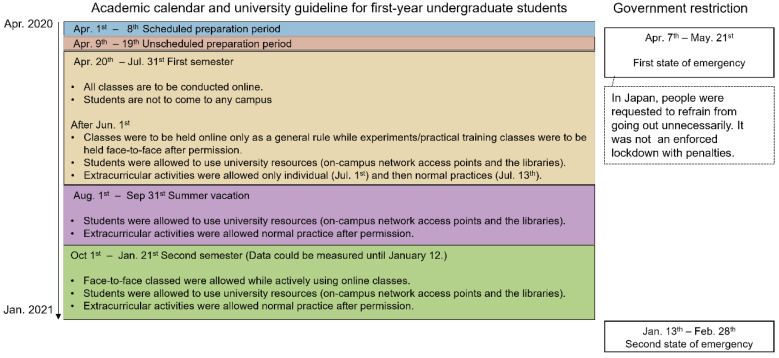
Summary of academic calendar with university guidelines for undergraduate students and Japanese government restrictions [34].

The entire recording period was divided into five periods according to the university’s academic calendar, which is expected to strongly influence the physical activity of university students as follows: (1) the scheduled preparation period (8 days), defined as the period of guidance that was set to occur immediately after the university’s entrance ceremony, regardless of the COVID-19 pandemic; (2) the unscheduled preparation period (11 days) accommodating the implementation of online classes owing to the COVID-19 pandemic; (3) the first semester (103 days) that ran from spring to summer; (4) the summer vacation (61 days) that followed; (5) and the second semester (104 days) that ran from fall to winter (Figure 1). A cross-correlation matrix with Pearson’s correlation coefficient was used to visualize relationships collectively between the median daily step count for each of the five periods. The diagonal components of the cross-correlation matrix were used to visualize the distribution of the median daily step counts for each of the five periods. If there was a correlation between the median daily step counts for each of the five periods, dimensionality reduction would be useful for subsequent clustering analysis and interpretation of the data. Therefore, a principal component analysis was performed to extract features from the median daily step counts for the same five periods. The number of principal components was determined by the relationship between the principal components and explained variance. A k-means clustering was performed using selected principal components and then evaluated using the silhouette value. The number of clusters was determined by the elbow method. The daily step counts with 7-day moving averages were classified based on the results of the k-means clustering. All data analysis was performed using MATLAB with Statistics and Machine Learning Toolbox (MATLAB R2021a).

## 3. Results

**Figure 2:**
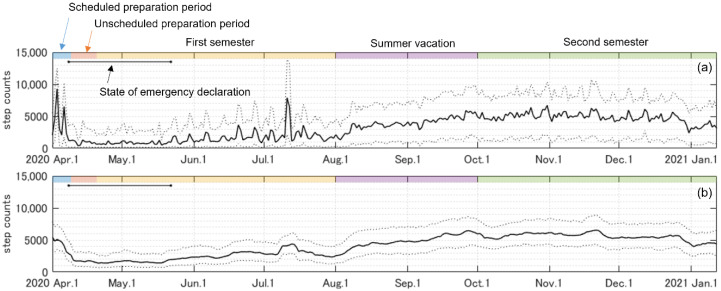
Time-series changes in the median daily step counts. (**a**) Median daily step counts (solid line) with 25% and 75% quantiles (dotted line) across 603 participants; (**b**) the 7-day moving average daily step counts (solid line) with 25% and 75% quantiles (dotted line).

Figure 2 shows the time-series changes in median daily step counts with 25% and 75% quantiles across 603 participants. Spikes in daily step counts were recorded at the beginning of April (9301 steps/day) and in mid-July (7903 steps/day), coinciding with the days when most participants had opportunities to visit the university (Figure 2a). The 7-day moving average daily step counts revealed a dramatic decrease during the first 10 days of April (Figure 2b). The daily step counts then maintained lower values during the state of emergency in Japan (from 7 April to 5 May), gradually increasing after the declaration that the state of emergency was lifted (Figure 2b).

**Figure 3:**
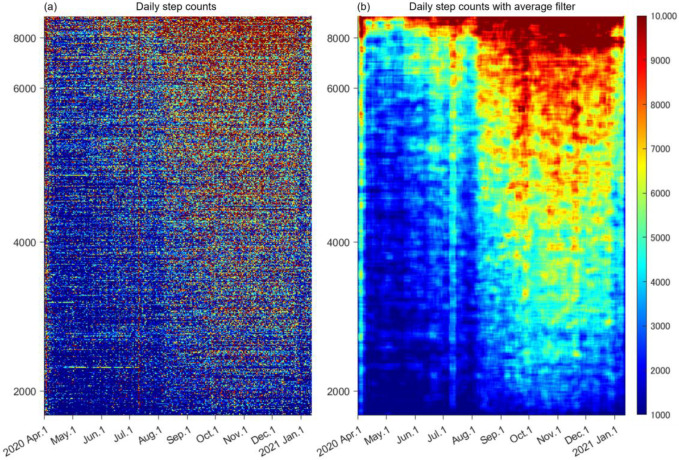
Daily step counts of 603 participants with moving averages. (**a**) Daily step counts represented as colormaps in descending order of median daily step counts from top to bottom. (**b**) Daily step counts after applying average filters with 10 participants × 7 days matrix.

In the colormap of the daily step counts (Figure 3a), each color dot represents the daily step counts of each participant. Participants who recorded over 8000 mean daily step counts across the target duration are shown with dark red dots, except for the state of emergency. With a decrease in the mean daily step counts (from top to bottom), the blue and dark blue dots increased across the target duration. The colormap of the daily step counts with moving average revealed that the timing of the transition from blue (<3000 steps/day) to yellow (>6000 steps/day) was delayed backwards as the mean daily step counts decreased from top to bottom (Figure 3b). The transition was small (from blue to light blue) in participants with lower mean daily step counts (<2000) (Figure 3b).

**Figure 4:**
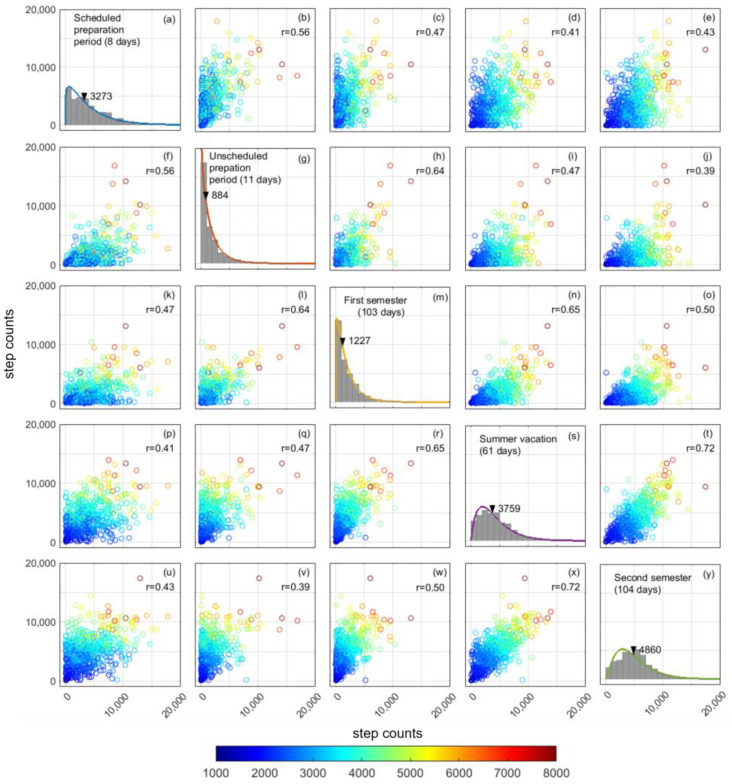
Cross correlation matrix of median daily step counts during five periods according to the academic calendar. Color of scatter represents the median step count of 603 participants during the entire recording period. Diagonal panels show the distribution of daily step counts during five periods (a, g, m, s, y). The arrow and value in histogram show the median value across participants during the period. Other panels show the relationship between two parameters.

**Figure 5:**
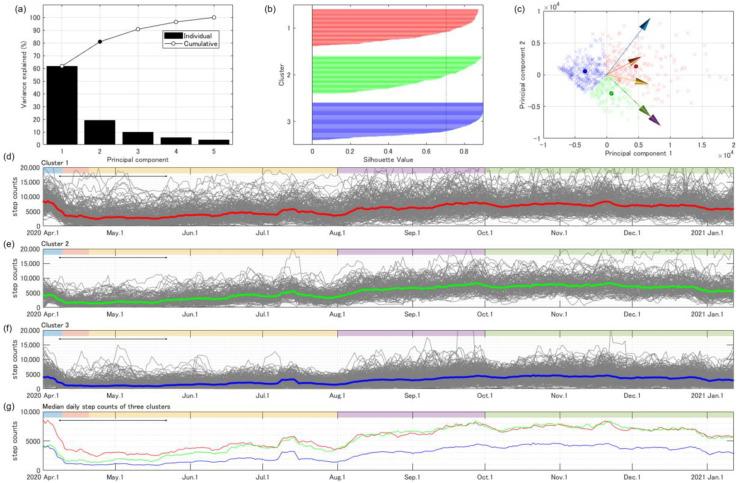
(**a**) Variance explained by the principal components extracted through the principal component analysis. (**b**) The accuracy of clustering was evaluated using the silhouette plot. The dotted line shows the mean silhouette value (0.704). (**c**) Biplot of scores of 603 participants (×) divided into three clusters (red, green, and blue) with the vectors of five original variables (arrows) on the plane determined by two principal components. The colors of the arrows correspond to the colors of each period in Figure 4. Circles show the mean value for each of the three clusters. (**d**–**f**) Time-series changes in the daily step counts with 7-day moving average in three clusters are represented with gray lines, and median values across participants are represented with red (cluster 1), green (cluster 2), and blue (cluster 3). (**g**) There is a unique pattern of time-series changes between 3 clusters.

The probabilistic distribution of the mean daily step counts during the five periods—divided according to the academic calendar—were fitted as a gamma distribution (Figure 4). The lowest median and mode were recorded during the unscheduled preparation period, with a strongly left-skewed distribution. The cross-correlation matrix revealed significant positive correlations for all combinations (*p* < 0.0001). Relatively strong correlations were observed among the first semester, summer vacation, and the second semester. In contrast, relatively weak correlations were observed for combinations related to scheduled and unscheduled preparation periods. Eighty percent variance was explained by the summation of the first two principal components, while the variance explained was only 60% when used only 1st principal component. The variance explained by each principal component after the third principal component was relatively small (less than 10%). (Figure 5a); therefore, the first two principal components were used in the k-means clustering. The number of clusters was set at three, and the silhouette values are presented in Figure 5b. Figure 5c (biplot) shows the distribution of the classified participants on the principal component plane and the vector representing the original variables that shows the daily step counts during five periods according to the academic calendar. The biplot revealed that the major difference between cluster 1 and 2 is the daily step count during the scheduled and unscheduled preparation periods, because the boundary of the two clusters was orthogonal to the vector representing both periods. The biplot also revealed that the major difference between two clusters, 2 and 3, is the daily step count during the summer vacation and second semester.

Figure 5d–f show the time-series changes in daily step counts with a 7-day moving average (gray) and the median value across participants in each of the three clusters (red, green, and blue). Clusters 1 (31% of participants) and 3 (47% of participants) show relatively high and low daily step counts, respectively. Cluster 2 (22% of participants) shows a relatively low daily step count from April to May. The daily step count then increases starting from June, as shown in clusters 1 and 2. Figure 5g shows the biplot of the scores of 603 participants divided into three clusters with loading vectors of five variables on the plane determined by two principal components.

## 4. Discussion

We investigated changes in university students’ physical inactivity during the COVID-19 pandemic according to their academic calendar by recording daily step counts through smartphone applications. To the best of our knowledge, this is the first study to report the daily step counts of university students across an extended period (287 days), including semester and vacation periods. The high degree of temporal resolution in this study, facilitated by smartphone application use, enabled the detection of continuous changes in the students’ daily step counts. The descriptive results revealed that the daily step counts were also influenced by periods on the academic calendar, as well as socially restrictive governmental measures in response to the COVID-19 pandemic. Additionally, clustering analysis demonstrated variations in the level of physical inactivity owing to the pandemic; for example, many inactive students did not fully recover in their daily step counts throughout the year. Physical activity should be promoted for inactive university students over the course of the whole semester during and after the COVID-19 pandemic.

**Figure 6:**
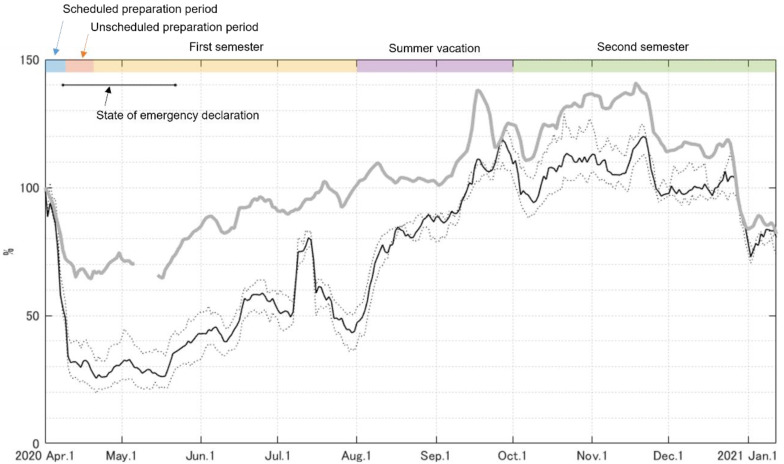
Apple Mobility Trend Reports with 7-day moving average (gray solid line) and median daily step counts with 7-day moving average (black solid line), both normalized by these data obtained on 1 April 2020.

The daily step counts decreased dramatically at the start of the state of emergency and unscheduled preparation periods in the beginning of April (Figure 2). In global survey data, the mean daily step counts in Japan showed a slow decrease from February to May; the minimum value (approximately 5000 steps/day) was observed in mid-April [25]. The baseline of mean daily step counts across Japanese participants (*n* = 20,386) was reported to be 6010 steps/day in a large-scale global survey using smartphone applications, conducted between July 2013 and December 2014 [21]. From these reports, the rate of decrease owing to the emergency declaration is estimated to be approximately 20%. In contrast, our data show that the reduction is estimated to be more than 60% before and during the state of emergency because the daily step count in nearly age-matched university students before the COVID-19 pandemic was reported to be approximately 7000 steps/day [35]. We also compared our daily step counts result (7-day moving average) with the Apple Mobility Trends Reports [36], which well reflects the number of walking bouts as an indicator of physical activity [37]. In this comparison report, we used the ‘walking’ mobility trend in Japan. The reduction rate shown in Apple Mobility Trend Reports during the state of emergency was approximately 20–30%, which corresponds to the decrease in daily step counts estimated from previous reports [21,25] (Figure 6). The daily step counts of university students in the current study demonstrated a greater reduction rate and slower recovery than the walking mobility trend in Japan (Figure 6). Thus, the physical activity of university students may be more susceptible to restrictive measures than indicated by the overall trend in Japan. The rate of decline in the physical activity of university students in the current study is estimated to be similar to that observed in countries where the government declared strict restrictive measures [14,24,29].

A possible cause of the great decline in the physical activity of university students is the shift to online classes during the COVID-19 pandemic. The contribution of conducting face-to-face classes on physical activity can be observed on days when most students were likely to commute to the university at the beginning of April (face-to-face guidance for first-year students) and middle of July (face-to-face educational event such as homeroom for first-year students) (Figure 2a). The university students are able to meet the recommended daily step counts (10,000 steps/day [18,19]) by commuting to school and/or walking around the campus when they have opportunities to attend university in person. This observation matches previous reports in which light physical activity decreased only in undergraduate students but not in others who attended or worked at the university [12]. Likewise, the results of a global survey report that the daily step count of a younger group was lower than that of the older group [14,29], as members of the younger group remained at home for longer periods during COVID-19 [29]. The levels of physical activity in university students are thus strongly related to the academic calendar, in addition to the restrictive governmental measures.

The probabilistic distribution of the median daily step counts across each period showed unique features (Figure 4a,g,m,s,y). The unscheduled preparation period was an extension of the scheduled period to lengthen the time allotted to shifting into online instructional mode. The unscheduled period started at almost the same time as the state of emergency in Japan. The distribution of the median daily step counts is left-skewed, indicating that most participants stayed at home, while a few participants maintained physical activity and were categorized into the active group (cluster 1) (Figure 5d). The left-skewed distribution was slightly corrected during the first semester, but the daily step count was low (Figure 4m). Thus, it seems that in this phase, participants who restarted physical activity gradually increased, with great individual variations in start date and daily step counts (Figure 3b), categorized as being in the recover group (cluster 2) (Figure 5e). We speculate that most participants did not take time for physical activities between managing online classes and homework during the first semester, even after the end of the state of emergency. Other participants, however, began to make time for physical activity. After the end of the first semester, the daily step counts increased during summer vacation (Figure 2 and Figure 3), and the left-skewed distribution was corrected by a reduction in low daily step counts (Figure 4s). In the second semester, the university adopted a hybrid of face-to-face and online classes. The median daily step counts reached 6000 steps/day (Figure 4y), which is the reported baseline of Japanese daily step counts on average [21], despite not all participants having opportunities to go to the university campus during the second semester. However, there were many students who remained at a lower level of daily step count, categorized in the inactive group (cluster 3), unaffected by the academic calendar. The low level of daily step counts could be due to the group being originally less active, or it could be due to the COVID-19 pandemic, or both. The World Health Organization (WHO) have recommended at least 150 to 300 min/week of moderate-intensity aerobic physical activity or at least 75 to 150 min/week of vigorous-intensity physical activity [38]. Walking at a pace of approximately 1000 steps per 10 min has been reported to be equivalent to moderate physical activity, and people are encouraged to achieve at least 3000 step/day during 30 min for 5 days in a week [39]. A long-term continuous monitoring of daily step count and promoting physical activity through mobile health (mHealth) devices and applications may be effective interventions during and after the COVID-19 pandemic, taking advantage of the high rate of smartphone ownership among university students [20,40,41,42,43,44]. We believe that mHealth is expected to become an effective tool when opportunities for commuting to university decrease due to the increase in online classes.

A disadvantage of using a smartphone application is the accuracy of recording step counts in free-living conditions. Smartphone applications enable the recording of continuous steps in the experimental condition where the target step count is set [45,46]. The accuracy of step counts, however, recorded in free-living conditions has been known to be underestimated, due to the non-recorded steps that occur when the participant does not hold the smartphone [47]. The major source of recorded daily step counts by smartphones is locomotive activity. In contrast, step counts cannot be recorded during household and sports activities without carrying smartphone, resulting in the underestimation of daily step counts in the free-living condition. The participants were recommended to carry their own smartphone with them as much as possible during daily life and exercises (walking and jogging). However, in this study, we were not able to determine the time spent wearing the smartphone and the location of the phone in the free-living condition, which have been reported to underestimate the daily step count [47]. Additionally, the model of iPhone and the version of operating system were not standardized in this survey. However, it has been reported that the difference in the model of iPhone and version of operating system does not critically affect the recorded value [48,49]. Therefore, we speculated that the major finding of this study is not impacted by the possible systematic underestimation of the daily step counts recorded by smartphone applications [45,46,47,48,49,50]. Although the bias in recorded daily step count due to the reduction in the accuracy of smartphone applications has been of concern [51,52], we believe that the use of a smartphone application was an appropriate method to describe the variation in the physical inactivity of university students during the COVID-19 pandemic from a macroscopic perspective [53].

## 5. Conclusions

Our study aimed to examine the variations in physical inactivity during the COVID-19 pandemic, with respect to the academic calendar of the participating students. The smartphone-based survey allowed the efficient monitoring of physical activity with high temporal resolution. Our data show that the daily step count was greatly influenced by the university academic calendar and governmental restrictive measures in response to the COVID-19 pandemic. Clustering analysis demonstrated that variations in the level of physical inactivity occurred, and there were inactive students (47%) who showed a low level of daily step count throughout the year. We suggest that the monitoring and promotion of physical activity is essential to preserve the health of inactive university students during/after the COVID-19 pandemic.

## Data Availability

The data analyzed in this manuscript will be made available from the corresponding author upon reasonable request.
